# Research on the Fused Deposition Modeling of Polyether Ether Ketone

**DOI:** 10.3390/polym13142344

**Published:** 2021-07-17

**Authors:** Ruoxiang Gao, Jun Xie, Jinghui Yang, Chaojie Zhuo, Jianzhong Fu, Peng Zhao

**Affiliations:** 1 The State Key Laboratory of Fluid Power and Mechatronic Systems, Zhejiang University, Hangzhou 310027, China; grx@zju.edu.cn (R.G.); jxie93@zju.edu.cn (J.X.); jinghuiy@zju.edu.cn (J.Y.); zhuocj@zju.edu.cn (C.Z.); fjz@zju.edu.cn (J.F.); 2 Key Lab of 3D Printing Process and Equipment of Zhejiang Province, Zhejiang University, Hangzhou 310027, China

**Keywords:** polyether ether ketone (PEEK), fused deposition modeling (FDM), design of experiment (DOE), warpage, tensile strength

## Abstract

As a special engineering polymer, polyether ether ketone (PEEK) has been used widely due to its excellent mechanical properties, high thermal stability, and chemical resistance. Fused deposition modeling (FDM) is a promising process for fabricating PEEK parts. However, due to the semi-crystalline property and high melting point of PEEK, determining appropriate process parameters is important to reduce warpage deformation and improve the mechanical properties of PEEK. In this article, the influence of raster angle and infill density was determined by single factor experiment, which are the two most important parameters. The results showed that samples with 0°/90° raster angle and 50% infill density had the best comprehensive properties in terms of warpage deformation, tensile strength, and specific strength. Subsequently, based on the results above, the effects of printing speed, nozzle temperature, platform temperature, raster width, and layer thickness were analyzed by orthogonal experiment. The results indicated that platform temperature had the greatest impact on warpage deformation while printing speed and nozzle temperature were significant parameters on tensile strength. Through optimization, warpage deformation of the samples could be reduced to almost 0 and tensile strength could increase by 19.6% (from 40.56 to 48.50 MPa). This will support the development of FDM for PEEK.

## 1. Introduction

Three-dimensional (3D) printing is a technology that forms solid parts by accumulating materials layer by layer [1,2]. Different from traditional machining processes, it can greatly reduce the waste of materials, and also makes it possible to manufacture parts with complex geometries. Compared with other additive manufacturing technologies, fused deposition modeling (FDM) has a wide range of molding materials, low cost, simple equipment operation and maintenance, and less pollution to the environment [3,4]. This technology has been widely used. At present, an important research direction of FDM technology is studying the effects of printing parameters on the properties of products. In addition, more and more new raw materials have been researched to print in order to improve the comprehensive properties. Liu et al. [5] developed a pellet 3D printer using pure Polycaprolactone (PCL) pellets for 3D printing. Esmi et al. [6] successfully prepared 3D printing filaments using polymethyl methacrylate (PMMA)/carbon nanotube (CNT)/Hydroxyapatite (Hap) nanocomposites. Polyether ether ketone (PEEK) is another typical example, which is a special engineering plastic. Compared with ordinary 3D printing materials, such as acrylonitrile butadiene styrene (ABS) and polylactic acid (PLA), PEEK has good mechanical properties, biocompatibility, heat resistance, and chemical resistance [7]. Complex parts manufactured by PEEK can not only be used for human implants [8,9], but also gradually replace metal materials in aerospace and other fields [10,11]. In addition to traditional injection molding process, FDM 3D printing has become a new development direction of PEEK manufacturing.

However, as a semi-crystalline polymer, PEEK will crystallize during the cooling process and produces molecular orientation, which is different from amorphous thermoplastics [12,13]. Besides, PEEK has a quite high melting point (343 °C) and needs to be heated to more than 380 °C during the modeling process, but conventional printers cannot reach such a high temperature. Compared to other materials, the printed PEEK parts are more likely to experience a greater thermal gradient and they are prone to warp and deform during the printing process, which seriously affects performance of the product. Thus, there are many challenges in the FDM process of PEEK. Researchers have carried out extensive studies. Valentan et al. [14] successfully developed a 3D printer for thermoplastic modelling, which was capable of directly producing PEEK parts, and tested mechanical properties of manufactured products. To control the nozzle temperature effectively, Liu et al. [15] combined PID control and fuzzy control and realized the accuracy of the nozzle control and rapid reaction. In order to improve the performance of the product, Deng et al. [16] studied the effects of printing speed, printing layer thickness, temperature, and filling on mechanical properties of PEEK. Yang et al. [17] studied the relationship between the nozzle temperature, crystallinity, and mechanical properties of PEEK material through a temperature-controlled 3D printing system. The results show the potential to control the crystallinity and mechanical properties by adjusting processing temperatures. To study the effects of FDM equipment specifications, Wang et al. [18] used finite element simulation method to analyze and optimize the flow channel in the nozzle, and printing parameters can be obtained preliminary. Several experiments were also performed to study the effects of parameters on the mechanical properties, microstructure, and surface quality of printed PEEK parts. Furthermore, to improve the mechanical properties of PEEK parts, Li et al. [19] explore the effect of multi-factor coupling, such as different printing temperatures, printing directions, printing paths, and layer thicknesses on tensile strength, bending strength, crystallinity, and grain size of FDM printed PEEK parts.

Previous researches have demonstrated the feasibility of FDM process PEEK. It can be found that most of them are concentrated on the mechanical properties, which are the most prominent characteristic of PEEK [20,21,22,23,24,25,26,27]. However, the effects of process parameters on warpage deformation have not been clearly defined. In terms of parameter selection, most studies selected only a few parameters. In FDM process, there are many factors that affect performance of the products. Thus, current research is obviously not sufficient and comprehensive. In addition, many researchers used single factor analysis methods for experimental design. This will help to understand the effects of each parameter. However, the significance of different parameters cannot be compared, and the mutual influence between the parameters was ignored.

Herein, this article selected tensile strength as the index of mechanical properties. Warpage deformation was also studied. As many parameters as possible were considered, including raster angle, filling density, printing speed, nozzle temperature, platform temperature, raster width, and printing layer thickness. Through the combination of full factor experiment and orthogonal experiment, the effects of different parameters are comprehensively studied to obtain the best process parameters.

## 2. Materials and Methods

### 2.1. Equipment and Materials

The equipment used in this experiment is a F430 3D printer, which was produced by CreatBot company (Zhengzhou, China). The X/Y resolution was 0.01 mm and the Z resolution was 0.015 mm. The filament used was K10 PEEK filament produced by Kexcelled (Suzhou, China). Some properties of PEEK material are listed in Table 1. Similar to injection molding, before printing, the PEEK filament needs to be dried in an oven at 120 °C for 4 h to prevent defects such as bubbles in the final samples [28,29]. In order to improve the reliability of the results, three samples are printed for each group of parameters. After printing, the samples will be placed in the printer’s 70 °C chamber for 1 h, and finally cooled.

### 2.2. Tests and Parameters

**Figure 1:**
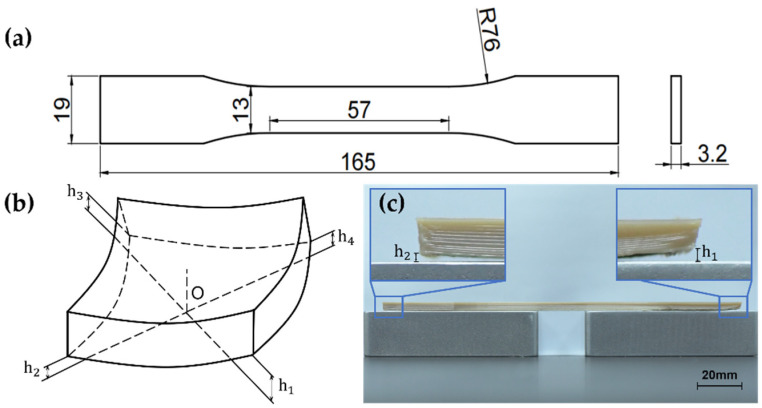
Tests and measurement of the product: (**a**) shapes and dimensions of the tensile sample; (**b**) measurement index to evaluate the warpage deformation; (**c**) warpage measurement methods based on the picture.

The mechanical properties of product are evaluated through tensile testing. Standard tensile samples are processed according to ASTM D638. Shapes and dimensions of the sample are shown in Figure 1a. Zwick/Roell Z020 (Berlin, Germany) testing machine was used for the test. The test speed was 5 mm/min. The test was carried out at a room temperature environment. After the tensile testing, the micromorphology of the fractured printed samples was observed using scanning electron microscopy (SEM, SU-3500, HITACHI, Tokyo, Japan).

There is no uniform standard for evaluating warpage deformation. Many researchers have proposed the measurement index of warpage deformation [30,31,32,33]. They all designed samples of specific shapes and proposed their own definitions, which are not universal. In this article, based on the shape of the standard tensile sample, we propose a measurement index, as shown in Figure 1b. This will be used as a comparison value for all printed samples of identical shapes. Measure the distance $h_{1},{\;h}_{2},{\;h}_{3},{\;\mathrm{and}\;h}_{4}$ between the four corners of the sample and the ground and find their average value as the warpage deformation of the entire model.

This article proposes a method to measure warpage based on images. As shown in Figure 1c, the sample is placed on gauge blocks of standard height (20 mm), and two picture of the sample and the blocks was taken from two directions. The height $h_{1},{\;h}_{2},{\;h}_{3},{\;\mathrm{and}\;h}_{4}$ was measured through the image processing software. The average value of $h_{1},{\;h}_{2},{\;h}_{3},{\;\mathrm{and}\;h}_{4}$ was an indicator of warpage deformation. In this method, the sample was in a free state, avoiding the errors caused by the traditional contact measurement method.

**Figure 2:**
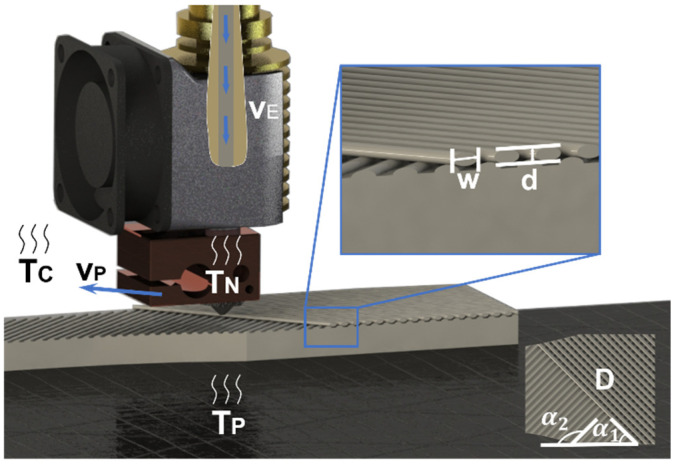
Schematic diagram of FDM 3D printing parameters: raster angle ($\alpha_{1}/\alpha_{2}$), infill density (D), layer thickness (d), raster width (w), nozzle temperature ($T_{N}$), platform temperature ($T_{P}$), chamber temperature ($T_{C}$), printing speed ($v_{P}$), extrusion speed ($v_{E}$).

The parameters of FDM can be divided into the following categories, structural parameters, temperature parameters, and speed parameters. Each category has different effects on the performance of printed parts. The structural parameters include raster angle, infill density, layer thickness, raster width, etc. Temperature parameters include nozzle temperature, platform temperature, chamber temperature, etc. Speed parameters include printing speed, extrusion speed, and so on. The definition of various parameters is shown in Table 2 and a schematic diagram of different parameters is shown in Figure 2.

### 2.3. Preliminary Experiment

#### 2.3.1. Raster Angle Optimization

Raster angle determines the direction of deposited materials, as Figure 2 shows. Researchers have found that raster angle has the greatest impact on tensile strength of 3D printing samples and has been the most extensively studied [34]. Therefore, this article first determined the optimal raster angle. Different raster angles of samples studied in the research were 0°/90°, 15°/105°, 30°/120°, and 45°/135°. The printing angles of two adjacent layers are perpendicular, which is a commonly used slicing method. Other parameters are set by referring to the experimental parameters of the previous researcher: printing speed was 40 mm/s, nozzle temperature was 420 °C, platform temperature was 160 °C, raster width was 0.4 mm, layer thickness was 0.1 mm, and infill density was 40%.

#### 2.3.2. Infill Density Optimization

Most of the previous researchers used 100% infill density, which can increase the strength of the samples, but the increase of thermal and force influence between deposition materials will lead to larger residual stress, resulting in warping deformation [35]. Singh et al. [36] indicates that reducing the infill density could prevent unwanted warping by subdividing the continuous stress generation. Studies have found that when the infill density reaches a certain level, the filling of the parts is sufficient, and the increase in filling has little effect on the strength improvement [16], and it will cause waste of materials, which are very expensive. Therefore, in order to achieve lightweight design, it is not necessary to use a 100% infill density. In this experiment, samples with an infill density of 20–100% were printed respectively and the tensile strengths were tested. The infill density is a programmed value. Other parameters followed the setting of the previous experiment.

### 2.4. Orthogonal Experiment

Through preliminary experiments, the influence of the two most important parameters can be determined. In addition to the above two structural parameters, there are many printing parameters that affect performance of the parts. The multi-factor test includes full-factorial design and fractional factorial design. The full factorial experiment can be used to test the combination of all parameters and consider the influence of these parameters comprehensively. However, the number of experiments is too large and the cost is high, so it is difficult to implement [37]. Orthogonal experimental design (OED) is a fractional factorial experiment design method that selects some representative points from the full factorial experiment based on orthogonality. The full factorial experiments could be understood by analyzing the fractional experiments [38]. Studies have shown that printing speed, nozzle temperature, platform temperature, raster width, layer thickness are important factors that affect tensile strength and warpage deformation of FDM samples. Thus, above five parameters were selected as influencing factors. In the commonly used orthogonal table, a $L_{18}\left(6^{1}\;3^{5}\right)$ orthogonal design table was selected for the experimental. It is a mixed-level design, that means the design has 18 runs, 1 factor with 6 levels and 5 factors with 3 levels. In the previous studies [39], printing speed is considered to be the most significant factor. Therefore, printing speed is initially selected as six levels, and other factors are at three levels. A blank column was set in the orthogonal table. There are no factors in the blank column and it just reflects the error caused by random factors, so the blank column is often called the error column in the analysis of variance. Besides, it is a comprehensive column that reflects the interaction that has not been investigated and other unknown influencing factors [40,41]. The levels of each factor are set as Table 3.

## 3. Results and Discussion

### 3.1. Preliminary Experiment

#### 3.1.1. Raster Angle Optimization

**Figure 3:**
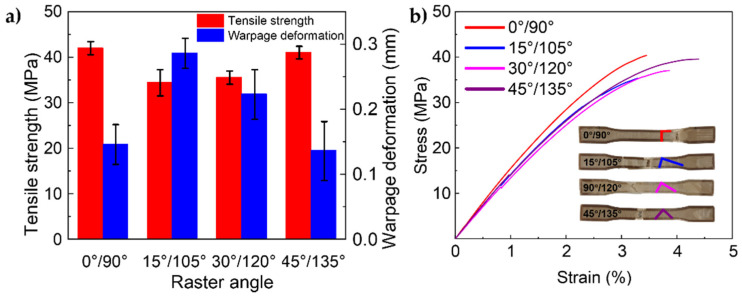
The results of the raster angle optimization. (**a**) tensile strength and warpage deformation; (**b**) stress-strain curve.

The tensile strength of different groups was obtained after the test. The results are shown in Figure 3a and the stress–strain curves are shown in Figure 3b. It can be seen that samples printed with raster angles of 0°/90° have the highest tensile strength. In addition, when the raster angle is 45°/135°, the sample structure is symmetrical and tensile strength is also relatively high. Tensile strength of the other two groups was significantly reduced. This result is consistent with the previous studies. The main reason is that when the samples’ raster angle is 0°/90°, the direction of deposition is the same as the direction of load, and the polymer chains prefer to be arranged along the orientation of these printing lines, so it could withstand greater tension without breaking. There will be shear between layers in other directions, which affects the bonding performance and reduces the tensile strength. In addition, the asymmetrical arrangement will result in uneven loading of the samples. Therefore, when raster angle is set to 15°/105° and 30°/120°, tensile strength will be significantly reduced compared to the other two groups.

Besides, the warpage deformation of different groups was measured. It can be seen form Figure 3a that samples with raster angles of 0°/90° and 45°/135° had a smaller warpage deformation. However, 0°/90° raster angle had the small error bar, which means the dimensional stability was better. In summary, a raster angle of 0°/90° was selected for the next phase of the experiment.

#### 3.1.2. Infill Density Optimization

**Figure 4:**
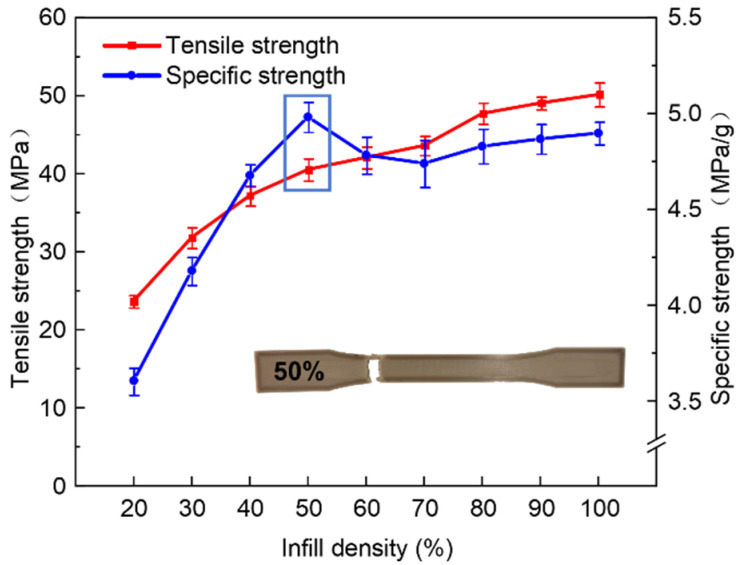
Effects of infill density on tensile strength.

In order to evaluate the effect of infill density more comprehensively, we define the ratio of tensile strength to mass of the sample as the specific strength, which was considered as an evaluation index. The curve of tensile strength and specific strength varying with infill density is shown in Figure 4.

There is no doubt that the larger the infill density, the higher the tensile strength. However, it can be seen that samples with 50% infill density show the highest specific strength. It can also be more intuitively reflected that the effect of improving the tensile strength is gradually decreasing when the infill density increases. Although reducing the infill density could make the tensile strength decrease, the samples could obtain greater specific strength and achieve a lightweight design. With comprehensive consideration, the infill density of 50% is selected as the optimal parameter for the next experiment.

### 3.2. Orthogonal Experiment

The results of the orthogonal experiment designed in Section 2.4 are shown in Table 4.

**Figure 5:**
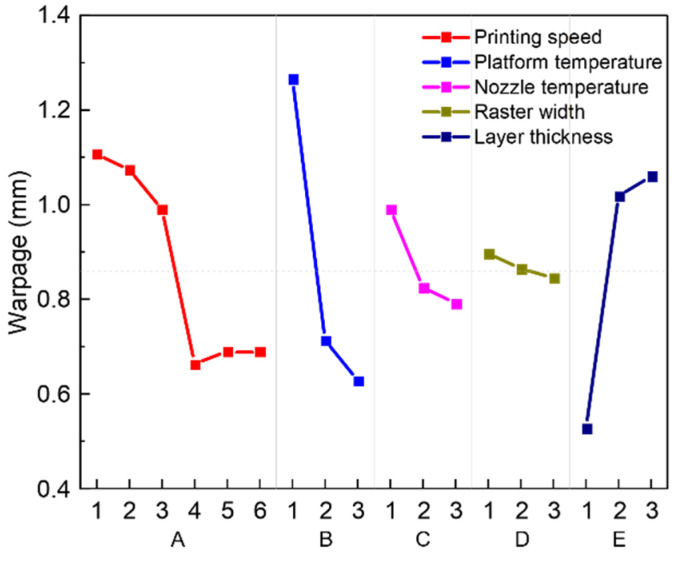
Main effects plot: relationship between warpage and factors with different levels.

Range analysis of the orthogonal experiment of warpage deformation was performed and the results are shown in Table 5, where K_1_, K_2_, K_3_, etc., respectively, represent the average value of each level under different factors, and $R^{b}$ represents the range between the maximum and minimum value of K_1_, K_2_, K_3_, etc. The value of $R^{b}$ can be used to judge the importance and the dominant degree of each factor. The larger the value, the greater the influence of the factor on the experimental results. It can be seen from Table 5 that, among the five main influencing factors, factor B has the largest range, that is, printing speed has the greatest impact on warpage deformation. The influence of the factor on warpage deformation followed the sequence: platform temperature > printing speed > layer thickness > nozzle temperature > raster width. Among them, the influence of raster width is not significant. The main effects plot of range analysis was shown in Figure 5, which can more intuitively show the degree of influence of different factors and different levels on the experimental results. We can see from Figure 5 that the best combination of process parameters was $A_{4}B_{3}C_{3}D_{3}E_{1}$, that is, the printing speed is 40 mm/s, the platform temperature is 160 °C, the nozzle temperature is 440 °C, the raster width is 0.6 mm, and the layer thickness is 0.1 mm.

Range analysis is a relatively simple method with a small amount of calculation, which can intuitively reflect the optimal parameter level, and initially determine the significance of each parameter. In addition to range analysis, the analysis of variance (ANOVA) is also a method for judging the significance of each factor [42]. ANOVA requires a lot of calculations, which take account in the influence of experimental errors, and can have more accurate results than range analysis. Taking warpage information as the observation index. The results of the analysis of variance are shown in Table 6. There are five important factors in the analysis of variance: the sum of squares of deviations (SS), degrees of freedom (DF), mean square error (MS), F-test statistic, and the *p*-value of the test statistic. These factors can be calculated by mathematical methods. In ANOVA, SS represents a measure of variation from the mean, which comes from the five actual factors, random factors, or errors. MS represents an estimate of the variance, which is calculated by dividing the corresponding SS by DF. The F value is a direct parameter. It is determined whether a factor is significant by comparing this value with a critical value in the F distribution table. If the F value is larger than the critical value, the factor is significant. The F value is calculated by dividing the MS of each factor by the MS of the error. The critical values in the F distribution table used for this orthogonal experiment are: F_0.1_ (2,2) = 9.0, F_0.05_ (2,2) = 19.0, F_0.01_ (2,2) = 99.0, F_0.1_ (5,2) = 9.29, F_0.05_ (5,2) = 19.30, F_0.01_ (5,2) = 99.25. It can also be tested by the *p* value. If the *p* value is less than the given α significance level (α = 0.001, 0.01, and 0.05), it indicates that the influence of different levels of this factor is significant [43,44]. In addition, the larger the F value, the smaller the *p* value, indicating that the impact of the factor is more significant. The influence of each factor on the observed could be calculated according to the ANOVA results. From Table 6, we can see that platform temperature had the most significant effect on the observed index, followed by layer thickness, printing speed, nozzle temperature and raster width. This sequence is consistent with the results of the range analysis.

**Figure 6:**
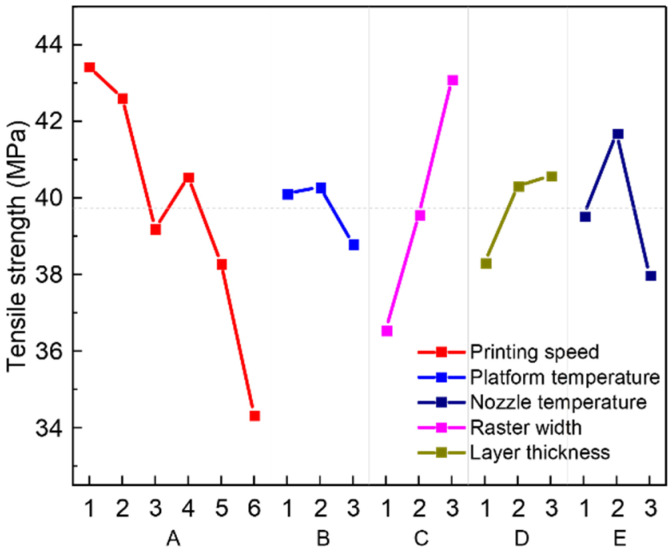
Main effects plot: relationship between tensile strength and factors with different levels.

The results of range analysis of each parameter on tensile strength are shown in Table 7. According to the values of $R_{b}$, the influence of each factor on tensile strength follows the sequence: printing speed > nozzle temperature > layer thickness > raster width > platform temperature. According to Figure 6, the process parameters should be $A_{1}B_{2}C_{3}D_{3}E_{2}$ to obtain the maximum tensile strength. Thus, the optimal process condition was: the printing speed is 25 mm/s, the platform temperature is 140 °C, the nozzle temperature is 440 °C, the raster width is 0.6 mm, and the layer thickness is 0.15 mm. The changing trend of a single factor is consistent with previous studies [45].

The results of ANOVA are shown in Table 8. The influence of each factor on tensile strength could be calculated according to the ANOVA results. According to the results, the influence of nozzle temperature is the most significant, followed by printing speed, layer thickness, raster width, and platform temperature. However, this result is different from the range analysis (printing speed has the most significant impact). Based on the previous comparison of the two methods, the results of ANOVA are more accurate to evaluate the significance of each factor.

**Figure 7:**
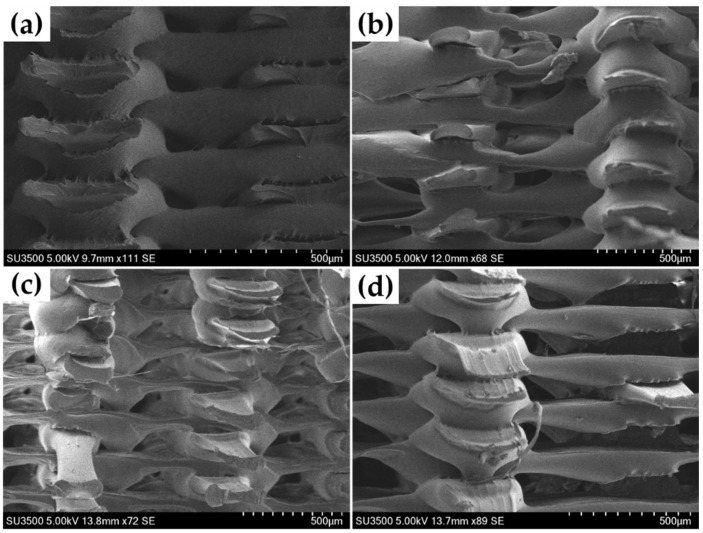
Representative SEM of fracture morphology of PEEK samples printed with different parameters. (**a**) Experiment No. 3; (**b**) Experiment No. 6; (**c**) Experiment No. 7; (**d**) Experiment No. 13.

It can be observed that there are obvious differences in the appearance and fracture mode of samples with different strengths. Samples with low tensile strength have obvious separations between layers after being stretched. This phenomenon shows that the bonding force between layers is too weak, which is also the main reason for their fracture. Due to the severe interlayer separation in the low-strength samples, only the fracture morphology of some samples with high strength and medium strength were observed by scanning electron microscopy (SEM). As shown in Table 4, samples in experiment No. 3 have the highest tensile strength, and their fracture morphology is shown in Figure 7a. Tt can be found that the adhesion between the layers of the high-strength samples is good, and the shape of the deposited filaments is uniform. Figure 7b–d shows the fracture morphology of other samples. It shows that the deposited filaments were obviously elongated, and there was a phenomenon of necking. The cross-sectional dimension of filament was not uniform during the deposition process. The raster size is normal only when there is deposited material at the bottom during deposition. When the raster hangs in the air during deposition (because the infill density was 50%), it will obviously become thinner. In addition, there were more holes and cracks between or within layers and the arrangement of the raster was messy.

The verification experiment was carried out with the optimal parameters ($A_{4}B_{3}C_{3}D_{3}E_{1}$ and $A_{1}B_{2}C_{3}D_{3}E_{2}$) obtained through the orthogonal experiment. The results showed that samples printed with parameters $A_{4}B_{3}C_{3}D_{3}E_{1}$ had the minimum warpage deformation, which was reduced to almost 0, and the tensile strength was 41.52 ± 1.84 MPa. In contrast, samples printed with parameters $A_{1}B_{2}C_{3}D_{3}E_{2}$ had the largest tensile strength of 48.50 ± 0.96 MPa, improved by 19.6% compared to the optimization result before preliminary optimization (samples printed with 0°/90° raster angle and 50% infill density had a tensile strength of 40.56 ± 1.41 MPa), while the warpage was 0.52 ± 0.08 mm. Both of them were better than the best results of the previous experiment group. The excellent performance shows the correctness and reliability of the orthogonal experiment. The final result is different from the nominal strength. This is because printed samples in our experiment used 50% infill density, which will have the highest specific strength and is of greater significance for lightweight design.

### 3.3. Discussion

#### 3.3.1. Printing Speed

Printing speed is the most important parameter affecting tensile strength. The group with printing speed of 25 mm/s had the highest tensile strength. As printing speed increases, the tensile strength shows a downward trend. At a lower printing speed, the sample had a larger filament width and higher strength [46]. Therefore, the strength of the overall sample would be higher. When printing speed increases, the printing efficiency could be improved, but residence time of the filament in nozzle flow channel is small, which is likely to cause incomplete melting and reduce fluidity. It will also cause uneven extrusion, weak interlayer bonding, and poor compactness, which will weaken mechanical properties.

When printing speed does not exceed 40 mm/s, the effect of printing speed on the amount of warpage is opposite to tensile strength, that is, the increase in printing speed leads to a decrease in the amount of warpage. This phenomenon is probably related to the temperature gradient within each layer. Due to the lower printing speed, it takes longer to complete the printing process for one layer. This leads to a larger temperature gradient in the layer and therefore higher warpage would appear [12]. In contrast, due to the higher deposition rate, the time taken to complete the deposition of a layer is shorter. This results in a smaller temperature gradient within the layer and therefore the warpage was lower. However, the amount of warpage starts to rise slowly when the printing speed exceeds 40 mm/s, and this may be due to the large residual stress caused by the excessive speed.

#### 3.3.2. Platform Temperature

The influence of platform temperature on tensile strength is not significant. The maximum difference in tensile strength between different levels of platform temperature is only 1.48 MPa. At present, few researchers have conducted research on the influence of platform temperature on mechanical properties. Tseng et al. [47] studied the coefficient of adhesion friction and found that high platform temperature during the printing process would cause the polymer layer to incompletely solidify and weaken the interlayer adhesion. This may cause a decrease in tensile strength.

In contrast, when the platform temperature increases, the warpage deformation is significantly reduced. Studies have found that there is an enhanced chain mobility of the deposited filament with higher temperatures [48,49]. Wang et al. [50] also found that, by increasing the temperature of the hot bed to a certain extent, the molecules can diffuse more sufficiently, thereby reducing defects such as voids, and the printed filament has a smoother appearance and demonstrates better adhesion. Therefore, the adhesion of the deposited material to the platform rises steadily and the warpage is reduced. In addition, warpage is related to the glass transition temperature [29,51]. When the platform temperature is raised to slightly higher than the glass transition temperature of the PEEK material ($T_{g}=1$43 °C), the adhesion between platform and first layer material could be significantly improved. Therefore, when the platform temperature is 160 °C, the samples could have the smallest amount of warpage deformation.

#### 3.3.3. Nozzle Temperature

Nozzle temperature of the low-strength samples was generally lower. When nozzle temperature increases, tensile strength gradually increases. This is because higher nozzle temperature can make the PEEK filament melt more sufficiently, improve the flow properties of the melt, and obtain a stable melting state. Temperature also has a certain effect on the level of crystallization [52]. The higher the temperature, the more crystallization. A high temperature nozzle could also be used as a heat source to have a heating effect on the solidified layer, which can make it in a semi-melted state and promote the adhesion of the filament and the solidified layer [19]. When the temperature is low, it is not fully heated, and the melting state is uneven, which leads to the deterioration of the performance of the extruded filament and reduces the tensile strength. Based on exactly this principle, Hu et al. [53] added a heat collector module and new high-power heater to the traditional nozzle to further increase the temperature in the forming area.

Warpage deformation tends to decrease with the increase of nozzle temperature. This is because, as nozzle temperature increases, the residual stress decreases, resulting in a decrease in warpage. However, a higher nozzle temperature could lead a higher temperature difference between extruded filament and printing chamber, which will result in a larger thermal gradient in the samples [54]. Therefore, it can be seen that, as the temperature increases, the tendency of warpage to decrease gradually slows down.

#### 3.3.4. Raster Width

The influence of raster width on tensile strength is also significant. As raster width increases, the tensile strength gradually increases. When the raster width is 0.6 mm, the average strength is the largest. This is because, with a larger printing raster width, the pressure in the melting chamber increases and the internal defects of the extruded filament are reduced, just like injection molding [55]. In addition, the adjacent contact area of the deposited materials between the layers increases and the adhesion becomes better, resulting in a higher strength.

In contrast, the influence of raster width on the amount of warpage is not significant, which is also consistent with the results of previous studies [56].

#### 3.3.5. Layer Thickness

As the thickness of the printed layer increases, the tensile strength first increases and then decreases. This may because if the layer thickness is too small, the distance between the nozzle and the deposited part is too small. This will cause the extruded filament to be squeezed and stretched. Defects will appear and ultimately resulting in a decrease in strength. When the layer thickness is too large, the additional force applied by the nozzle is lacking during the deposition of the filament, which affects the bonding effect between the extruded filament and the deposited part, and the overall tissue compactness becomes worse. Layer thickness can also indirectly affect the width of deposited filaments. In addition, there is a more obvious step effect, which causes uneven stress to a certain extent. According to the research of Wang et al. [18], when the layer thickness is greater than half of the nozzle diameter, the mechanical properties of the printed sample will be significantly reduced.

The amount of warpage deformation gradually increases when the thickness of the printed layer increases. This is because when the layer thickness is smaller, the cross-sectional area of a single raster is smaller, and the shrinkage is reduced. When the layer thickness increases, the volume of the single raster also increases, and the shrinkage will be greater, which increases the warpage deformation.

In summary, we must ensure that the optimal parameters of different indexes are not the same. So, in a practical production process, the parameters should be selected according to the different performance requirements. For example, if the demand for product strength is greater, parameters $A_{1}B_{2}C_{3}D_{3}E_{2}$ should be selected. On the contrary, parameters $A_{4}B_{3}C_{3}D_{3}E_{1}$ should be selected when accurate appearances and product shapes are preferred.

## 4. Conclusions

In this article, the effects of printing parameters for the FDM process on the warpage deformation and tensile mechanical strength of PEEK were investigated. Different process parameters were considered, including raster angle, infill density, printing speed, nozzle temperature, platform temperature, raster width, and layer thickness. The following conclusions can be drawn:

(1) Samples with a raster angle of 0°/90° had the greatest tensile strength. Besides, the warpage deformation was stable and relatively small compared with samples printed with other raster angles. Further, 50% infill density could make the samples reach the highest specific strength, which is defined as the tensile strength per unit mass. The warpage deformation of the part was also reduced with the decrease of infill density.
(2) Orthogonal experiment results showed that platform temperature is the most important parameter that affects warpage deformation. However, printing speed and nozzle temperature had more significant effects on tensile strength. The optimal parameter settings can be obtained. After the verification experiment of two different parameter groups, the warpage deformation of printed samples could be almost reduced to 0, while the largest tensile strength could reach 48.5 MPa.
(3) Increasing nozzle temperature could not only decrease warpage deformation, but also increase tensile strength. However, increasing printing speed and platform temperature would lead to samples with smaller warpage deformation while the tensile strength would decrease. In addition, the influence of raster width and layer thickness on the two indexes was also not the same.

It is difficult to meet the optimal conditions at the same time. So, the balance of two indexes must be considered in practical production. In addition, there are some performance requirements, such as surface roughness and dimensional accuracy. Therefore, multi-objective optimization is also needed. In terms of lightweight design, this article only considered the impact of infill density, which is a programmed value. The real cross-section and topological structure design could be taken into account in the future work. Besides, instead of starting with process optimization, methods to reduce warpage are worth studying, such as treating the bed with chemical solutions and improving the properties of PEEK filaments through fillers.

## Figures and Tables

**Table 1:** Some properties of PEEK material used in this research.

Properties	Value
Glass-transition temperature	143 °C
Melting temperature	343 °C
Tensile strength	70–80 MPa
Breaking elongation	6%
Bending strength	120 MPa
Bending modulus	2.6 GPa
Density	1.28 g/cm^3^

**Table 2:** The definition of various parameters.

Parameters		Description
Structure	Raster angle	The angle between the deposited material and the boundary.
	Infill density	Material percentage filling the component volume.
	Layer thickness	The thickness of the layer deposited.
	Raster width	The width of the deposited material.
Temperature	Nozzle temperature	The temperature of the extruder.
	Platform temperature	The temperature of the hot plate.
	Chamber temperature	The temperature of the 3D printer chamber.
Speed	Printing speed	The velocity of the material deposition.
	Extrusion speed	The velocity of the filament extrusion.

**Table 3:** Factors and Levels of the Orthogonal Experiment.

Level	Parameter				
	Printing Speed (mm/s)	Platform Temperature (°C)	Nozzle Temperature (°C)	Raster Width (mm)	Layer Thickness (mm)
	A	B	C	D	E
1	25	120	400	0.4	0.1
2	30	140	420	0.5	0.15
3	35	160	440	0.6	0.2
4	40				
5	45				
6	50				

**Table 4:** Results of the Orthogonal experiment.

Experiment No.	A	B	C	D	E	Blank	Warpage Deformation (mm)	Tensile Strength (MPa)
1	1	1	1	1	1	1	1.56 ± 0.10	38.23 ± 0.57
2	1	2	2	2	2	2	0.81 ± 0.04	45.20 ± 1.03
3	1	3	3	3	3	3	0.95 ± 0.15	46.82 ± 0.98
4	2	1	1	2	2	3	1.73 ± 0.27	44.23 ± 1.89
5	2	2	2	3	3	1	1.29 ± 0.20	41.31 ± 1.87
6	2	3	3	1	1	2	0.20 ± 0.05	42.28 ± 1.68
7	3	1	2	1	3	2	1.48 ± 0.11	36.94 ± 0.88
8	3	2	3	2	1	3	0.57 ± 0.14	44.06 ± 0.76
9	3	3	1	3	2	1	0.92 ± 0.19	36.61 ± 2.43
10	4	1	3	3	2	2	1.19 ± 0.14	46.49 ± 1.44
11	4	2	1	1	3	3	0.68 ± 0.14	34.83 ± 1.99
12	4	3	2	2	1	1	0.12 ± 0.04	40.34 ± 1.71
13	5	1	2	3	1	3	0.51 ± 0.05	39.20 ± 1.59
14	5	2	3	1	2	1	0.72 ± 0.14	43.25 ± 1.91
15	5	3	1	2	3	2	0.84 ± 0.09	32.40 ± 1.06
16	6	1	3	2	3	1	1.12 ± 0.16	35.64 ± 0.74
17	6	2	1	3	1	2	0.21 ± 0.04	33.05 ± 1.32
18	6	3	2	1	2	3	0.74 ± 0.15	34.34 ± 2.22

**Table 5:** Range analysis of the orthogonal experiment (warpage deformation).

Factor	A	B	C	D	E
$K_{1}$ (mm)	1.1067	1.2650	0.9900	0.8967	0.5283
$K_{2}$ (mm)	1.0733	0.7133	0.8250	0.8650	1.0183
$K_{3}$ (mm)	0.9900	0.6283	0.7917	0.8450	1.0600
$K_{4}$ (mm)	0.6633				
$K_{5}$ (mm)	0.6900				
$K_{6}$ (mm)	0.6900				
$R^{b}$ (mm)	0.4433	0.6367	0.1983	0.0517	0.5317
Rank	3	1	4	5	2

**Table 6:** Analysis of variance for the orthogonal experiment (warpage deformation).

Source	DF	SS	MS	F	P
A	5	0.65778	0.131556	1.31	0.407
B	2	1.43381	0.716906	7.16	0.048
C	2	0.13534	0.067672	0.68	0.559
D	2	0.00814	0.004072	0.04	0.961
E	2	1.04901	0.524506	5.24	0.076
Error	4	0.40049	0.100122		
Total	17	3.68458			

**Table 7:** Range analysis of the orthogonal experiment (tensile strength).

Factor	A	B	C	D	E
$K_{1}$ (MPa)	43.42	40.12	36.56	38.31	39.53
$K_{2}$ (MPa)	42.61	40.28	39.56	40.31	41.69
$K_{3}$ (MPa)	39.2	38.8	43.09	40.58	37.99
$K_{4}$ (MPa)	40.55				
$K_{5}$ (MPa)	38.29				
$K_{6}$ (MPa)	34.34				
$R^{b}$ (MPa)	9.07	1.48	6.53	2.27	3.70
Rank	1	5	2	4	3

**Table 8:** Analysis of variance for the orthogonal experiment (tensile strength).

Source	DF	SS	MS	F	P
A	5	161.793	32.359	7.62	0.036
B	2	7.965	3.983	0.94	0.463
C	2	128.278	64.139	15.11	0.014
D	2	18.435	9.217	2.17	0.230
E	2	41.385	20.692	4.87	0.085
Error	4	16.983	4.246		
Total	17	374.837			

## Data Availability

The data presented in this study are available on request from the corresponding author.
